# Pollen-mediated gene flow from transgenic to non-transgenic switchgrass (*Panicum virgatum* L.) in the field

**DOI:** 10.1186/s12896-017-0363-4

**Published:** 2017-05-02

**Authors:** Reginald Millwood, Madhugiri Nageswara-Rao, Rongjian Ye, Ellie Terry-Emert, Chelsea R. Johnson, Micaha Hanson, Jason N. Burris, Charles Kwit, C. Neal Stewart

**Affiliations:** 1 Department of Plant Sciences, University of Tennessee, 252 Ellington Plant Sciences, 2431 Joe Johnson Dr., Knoxville, TN 37996 USA; 2 Department of Biology, New Mexico State University, PO Box 30001, MSC 3AF Las Cruces, NM USA; 3 Department of Forestry, Wildlife and Fisheries, University of Tennessee, 274 Ellington Plant Sciences, 2431 Joe Johnson Dr., Knoxville, TN 37996 USA

**Keywords:** Switchgrass, Bioenergy, Hybridization, Gene flow, Pollen dispersal, Transgenic, Orange-fluorescent protein, Biosafety

## Abstract

**Background:**

Switchgrass is C_4_ perennial grass species that is being developed as a cellulosic bioenergy feedstock. It is wind-pollinated and considered to be an obligate outcrosser. Genetic engineering has been used to alter cell walls for more facile bioprocessing and biofuel yield. Gene flow from transgenic cultivars would likely be of regulatory concern. In this study we investigated pollen-mediated gene flow from transgenic to nontransgenic switchgrass in a 3-year field experiment performed in Oliver Springs, Tennessee, U.S.A. using a modified Nelder wheel design. The planted area (0.6 ha) contained sexually compatible pollen source and pollen receptor switchgrass plants. One hundred clonal switchgrass ‘Alamo’ plants transgenic for an orange-fluorescent protein (OFP) and hygromycin resistance were used as the pollen source; whole plants, including pollen, were orange-fluorescent. To assess pollen movement, pollen traps were placed at 10 m intervals from the pollen-source plot in the four cardinal directions extending to 20 m, 30 m, 30 m, and 100 m to the north, south, west, and east, respectively. To assess pollination rates, nontransgenic ‘Alamo 2’ switchgrass clones were planted in pairs adjacent to pollen traps.

**Results:**

In the eastward direction there was a 98% decrease in OFP pollen grains from 10 to 100 m from the pollen-source plot (Poisson regression, F1,8 = 288.38, *P* < 0.0001). At the end of the second and third year, 1,820 F_1_ seeds were collected from pollen recipient-plots of which 962 (52.9%) germinated and analyzed for their transgenic status. Transgenic progeny production detected in each pollen-recipient plot decreased with increased distance from the edge of the transgenic plot (Poisson regression, F1,15 = 12.98, *P* < 0.003). The frequency of transgenic progeny detected in the eastward plots (the direction of the prevailing wind) ranged from 79.2% at 10 m to 9.3% at 100 m.

**Conclusions:**

In these experiments we found transgenic pollen movement and hybridization rates to be inversely associated with distance. However, these data suggest pollen-mediated gene flow is likely to occur up to, at least, 100 m. This study gives baseline data useful to determine isolation distances and other management practices should transgenic switchgrass be grown commercially in relevant environments.

**Electronic supplementary material:**

The online version of this article (doi:10.1186/s12896-017-0363-4) contains supplementary material, which is available to authorized users.

## Background

Switchgrass (*Panicum virgatum* L.) is a perennial C_4_ bunchgrass that is native to North America and is widely distributed from Canada to Mexico where it occasionally reaches “common” status in certain prairies [1–4], marshes [5], conservation reserve program settings [6, 7], and along roadsides and waste places [8, 9]. Elsewhere, it is typically not a large component of natural areas [10, 11] and is often found in much lower densities than those grown in agronomic settings. Traditionally switchgrass has been grown as a forage crop [12, 13], and because it is highly adaptable, it has served a variety of purposes including conservation, wildlife habitat, prairie restoration, erosion control, and in ornamental gardens [12, 14, 15]. Owing to its high biomass yield and suitability for marginal land, switchgrass has been the target of extensive development as a dedicated lignocellulosic biofuel feedstock [16]. With the current drive to meet U.S. government-mandated benchmarks for energy production (16 billion gallons (1 gallon = 3.79 L) cellulosic biofuel by 2022; [17]), large-scale agronomic plantings of dedicated biofuel crops, including switchgrass, are projected to increase significantly through 2022 [14, 18, 19]. Although switchgrass research and development of conventional cultivars has significantly expanded over the last 25 years [20], extensive research efforts have been undertaken to produce transgenic switchgrass cultivars with increased biomass, enhanced saccharification efficiency, and modified lignin biosynthesis for improved biofuel properties [21–30]. Indeed, genetic engineering appears to be the only route to overcome biomass recalcitrance and sustainably improve biomass and biofuel production in tandem [31–33].

Although genetic engineering provides an opportunity to increase biomass yields and efficiencies in switchgrass production, transgenes from large-scale switchgrass plantings could escape to (1) non-transgenic switchgrass that might be used for livestock feed or other future uses, and (2) wild relatives in the landscape matrix [14, 31, 34]. Switchgrass is also being engineered for abiotic and biotic resistance traits (e.g. drought and salt tolerance, as well as pathogen resistance) [35], which could lead to increased distribution as well as shifted ecological niches if introgressed in conspecific or wild relative lineages. Switchgrass already exhibits a number of characteristics associated with invasiveness [36, 37]. These include C_4_ photosynthesis, a long canopy duration, few known pests and diseases, rapid growth in the spring (growing season), below-ground partitioning of nutrients in the fall (dormant season), and high water-use efficiency [38]. The potential repercussions from habitat shifts include conservation concerns, with two different extremes being (1) the subsequent rapid spread of a transgene throughout the wild population and enhancing the invasiveness of the recipient population, or (2) extinction of the wild population [14, 38–42]. The likely route of transgene escape from switchgrass is through pollen-mediated gene flow [31]. It is well documented that wind-mediated pollen- and gene flow readily occurs in many grasses (reviewed by Kausch et al. [34]), and transgene escape and establishment has been documented with herbicide resistant creeping bentgrass (*Agrostis stolonifera* L.) [43–45]. Switchgrass shares similar characteristics (perennial, wind-pollinated, obligate outcrossing species) with creeping bentgrass, and considering field sites in the U.S. would be located near wild conspecifics and congeners, it is conceivable that pollen-mediated gene flow would occur [14]. Yet to date relatively few studies have been published concerning switchgrass pollen biology and dispersal [46, 47]. The dearth of gene flow data, especially in the U.S. is largely to regulatory conditions: all regulated field trials to date require panicles to be removed at the R0 stage, obviating the ability to assess transgenic pollen movement and hybridization from transgenic pollen donors on non-transgenic pollen recipients.

It is imperative that we increase our existing knowledge on the drivers of transgene spread to satisfy imminent regulatory and conservation concerns regarding future transgenic bioenergy crops; indeed, stewardship of resources is a major reason. For switchgrass, we know little about pollen spread. To address the knowledge gap, we obtained release permits (11-007-106r and 13-046-104r-a1) through the United States Department of Agriculture’s Biotechnology Regulatory Services for the release of transgenic switchgrass expressing an orange fluorescent protein gene and a hygromycin resistance marker gene in order to perform a small multi-year pollen dispersal and cross-pollination field study. The study site was located in a secluded area surrounded by forest on all sides. Although the study was small in size, this experiment provided opportunity to gather much needed empirical data on transgenic switchgrass pollen dispersion and pollination distances under field conditions over a 3-year period in which permit conditions allowed plants to flower and set seed. These findings will aid in management practices for future cultivation of transgenic switchgrass, will be useful to both biosafety regulators and conservation biologists, and may also serve as a model for the incorporation of transgenic cultivars of other bioenergy feedstocks into the landscape.

## Results

### Transgenic lines and strategy

We used ‘Alamo’ switchgrass, which is a viable lowland cultivar grown in the southeastern U.S. While switchgrass is known to have various ploidy levels, lowland varieties are diploidized ancient allotetraploids. Our strategy for estimating gene flow in switchgrass was based on using a relatively large number of pollen-source to create a windborne ‘pollen cloud’ for pollinate pollen recipient plants at various distances and directions. Given that switchgrass is putatively self-incompatible, our goal was for the vast majority of pollen produced by source plants to harbor the transgene. For that reason, we chose transgenic switchgrass lines containing multiple T-DNA insertions to ensure that the vast majority of pollen grains were transgenic. In addition, we also chose lines for which the orange fluorescent protein (OFP) marker gene was clearly detectable in pollen and vegetative tissues [48]. Multiple transgenic events were screened for T-DNA copy number and OFP fluorescence [48] through Southern blot analysis and epifluorescent microscopy (Additional files 1 and 2). Based on the above selection criteria, transgenic lines ‘1–4’ and ‘2–9’ were chosen as pollen donors in the field experiment. Line 1–4’ possessed two transgene copies and line ‘2–9’ had six copies (Additional file 1), and exhibited OFP fluorescence when screened (Additional file 2). Moreover, we observed that practically every pollen grain was clearly OFP-positive, which ensured that the vast majority of pollen grains were transgenic. Assuming disomic inheritance and no association of different copies of the transgenes, 25% and 1.6% of non-transgenic pollen grains are expected in lines “1–4” and “2–9”, respectively (based on two and six transgene copies). Considering about 1/3 and 2/3 of the former and the latter plants in pollen donor population, the overall frequency of non-transgenic pollen should be about 9.4%. Since transgenic plants are expected to fertilize homozygous recessive (for the transgenic locus) pollen recipient plants, the same phenotypic frequencies are expected in hybrid seedlings. Of course, these are expectations on average, and it is possible that the apparent homogeneity of OFP pollen fluorescence observed in Additional file 2 is from sampling error, which is to say, we could have missed the non-transgenic pollen in the survey. Alternatively, the happenstance of transgene integration could have led to the observed pollen fluorescence distribution, which was apparently higher than the ~90% expected transgenic from the discussion above. We consider both scenarios in the Discussion section.

### Plant growth and productivity

To fully assess the growth conditions, soil parameters such as water pH, buffer value and other micro nutrients were recorded, and the soil fertility was considered to be low-to-marginal (Additional file 3). The pollen-source plot mean tiller number (44 per plant) and height (107.5 cm) were comparable to plants in pollen-recipient plots (Table 1). The pollen-recipient plots had a wide range in means for both tiller number (7–80 tillers per plant) and plant height (74.0–208.6 cm). Above-ground biomass was measured for the 2014 season only (Table 1), and the mean weight of pollen-source plants (2.6 g per plant) was low compared to the pollen-recipient plots means (1.0–70.7 g per plant).

**Table 1:** Growth characteristics of pollen-source and pollen-receptor switchgrass plants grown under field conditions

Field plot	Tiller number	Height (cm)	Above-ground biomass (g)
Pollen source	44 ± 13.0	107.5 ± 11.0	26
North 10	29 ± 13.0	128 ± 389	10
North 20	27 ± 10.5	142.9 ± 22.5	78
South 10	18 ± 8.4	170.5 ± 7.5	52
South 20	10 ± 2.5	103.2 ± 14.4	24
South 30	24 ± 5.0	132.7 ± 21.3	96
West 10	19 ± 10.9	182.2 ± 4.1	81
West 20	7 ± 0.5	90.8 ± 13.0	14
West 30	21 ± 6.5	95.9 ± 15.6	48
East 10	24 ± 1.5	134.6 ± 24.1	24
East 20	19 ± 0.5	90.2 ± 23.5	10
East 30	15 ± 0.5	96.2 ± 21.7	18
East 40	24 ± 8.0	74.0 ± 10.0	16
East 50	23 ± 7.5	152.4 ± 7.6	130
East 60	32 ± 9.4	167.3 ± 11.6	87
East 70	48 ± 0.5	121.9 ± 26.7	94
East 80	70 ± 44.3	208.6 ± 1.1	707
East 90	67 ± 40.3	189.9 ± 7.3	404
East 100	80 ± 33.5	146.1 ± 32.4	464

Numbers for tillers, height, and above ground biomass are average per plant (means ± SE). Tiller number and height were recorded for both the 2013 and 2014 seasons. Measures for above-ground biomass were from the 2014 field season

### F_1_ progeny analysis

A total of 1,820 F_1_ seeds were collected from pollen-recipient plants and after cold-treatment, 962 germinated (52.9%). The total number of F_1_ seedlings analyzed for both the 2013 and 2014 seasons was 962, and a total of 204 F_1_ seedlings were deemed to be OFP positive then analyzed via OFP-gene specific PCR with congruent positive results. There were many seeds that did not germinate, which were not included in the final analysis as transgenic positive or negative. Nonetheless, most of these non-germinating seeds appeared to be small and pale with no detectable OFP-fluorescence in the seed; we deemed them to be non-viable. The number of F_1_ seedlings analyzed per pollen-receptor plant in the east direction was 717; 66 from the west, 91 from the south, and 88 from the north. Additional file 4, a sample gel from the PCR analysis, and Additional file 5, the summary Table S2, show that a portion of the progeny from pollen-recipient plants are not transgenic.

**Fig. 1:**
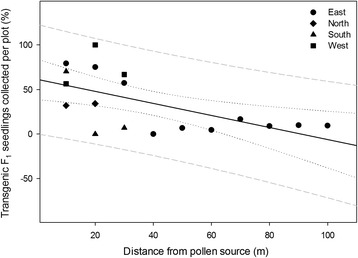
Relationship between transgenic F_1_ switchgrass seedlings collected from individual pollen-recipient plots and distance from the transgenic switchgrass pollen-source plot (Poisson regression, F1,15 = 12.98, *P* < 0.003). Pollen-recipient plots were located at 10 m intervals from the pollen-source and planted in the four cardinal directions. Seed were collected from the field site at the end of the 2013 and 2014 growing seasons

The east direction represented the long radius of the pollen-receptor plots as well as the direction of the prevailing wind. When normalized for pollen-receptor tiller number (Table 1), data indicated the number of F_1_ progeny collected from pollen-receptor plots to be inversely related to distance from the pollen-source plot (Fig. 1; Poisson regression, F1,15 = 12.98, *P* < 0.003). From the east direction, the majority of transgenic progeny were collected between 10 and 50 m plots (60.6%) compared to the remaining 40–100 m plots (39.4%). In addition, a greater than seven-fold decrease in transgenic progeny percentage was observed from the 10 m (79.2%) to 100 m (9.3%) plots. The percentages of transgenic F_1_ seedling observed in the west (10–30 m; 56.5–66.7%), south (10–30 m; 70.2–6.8%), and north (10–20 m; 32.0–34.2%) directions were similar to those observed in the east direction at similar distances (Additional file 5).

### Pollen dispersal

**Fig. 2:**
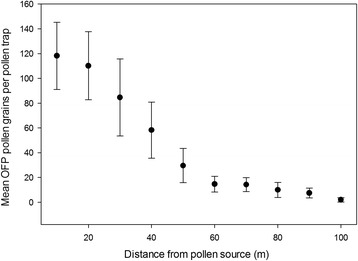
Average number of orange fluorescent protein (OFP)-tagged switchgrass pollen grains detected as a function of distance (Poisson regression, F1,8 = 288.38, *P* < 0.0001) under field conditions. Pollen grains were sampled from the eastward direction twice in 2012 and three times during the 2013 growing season (bars represent ± standard error of mean)

For all years, the Alamo 2 clonal type plants located in the pollen recipient plot began flowering in mid-July, while the ST1 clonal type plants in pollen-donor plots began booting the last week of July. Anthesis for both plant types overlapped starting in early August and continued until the last week of September of each growth season. During the pollen sampling period, the average wind speed was 5.2 km/h, the average dew point was 15.7 °C, the average temperature was 26.3 °C and the average humidity was 52.7% (Additional file 6) at the Oliver Springs study site (Additional file 7). OFP-tagged pollen counts collected from the eastward radius (direction of the prevailing wind) decreased as distance increased from the pollen-source plot (Fig. 2; Poisson regression, F1,8 = 288.38, *P* < 0.0001). These results indicate that the source pollen is capable of traveling at least 100 m, albeit at low frequency (ca. 0.01% of all switchgrass pollen from the source plants). More importantly, OFP-tagged pollen counts also declined with increased distance to the west (*R* ^2^ = 0.930), north (*R* ^2^ = 0.990), and south (*R* ^2^ = 0.993) directions from the source plants (Additional file 8). When OFP-tagged pollen counts (within 30 m) were compared across all the directions, the means of OFP-tagged pollen observed were highest in the eastward direction (104.3) as compared to the west (32.5), south (32.2) and north (29.4). The mean OFP-tagged pollen counts from 10 to 30 m away (104.3) from the source plants in the eastward direction were five-fold higher than mean pollen counts from 40 to 100 m (19.4).

## Discussion

The results presented here suggest that for switchgrass the majority of cross-pollination and transgenic pollen dispersal occur at short distances up to 50 m from the pollen source; however, a significant amount of pollen was detected at 50–100 m (Figs. 1 and 2). In addition, in the outermost pollen-receptor plot, 9.3% of the F_1_ seedlings recovered were transgenic. Our study is the first to record empirical data on switchgrass pollen dispersal and pollination distances in the field.

Transgenic cross-pollination percentages presented here in switchgrass as a function of distance from a transgenic pollen source are consistent with findings from other experimental studies concerned with transgene flow from a transgenic pollen source [49–59]. While the breeding systems and spatial scales involved in such studies vary widely, the primary conclusion is that the majority of transgenic cross-fertilization with nontransgenic counterparts occurs at short distances. In addition, studies addressing wind-pollinated grasses and having the largest spatial scales indicated transgenic cross-pollination percentages of 0.08% at 25 m from a transgenic source, and 0.006% at 250 m in maize [56], and levels declining to < 1% at 150 m in tall fescue [52]. In our study, transgenic cross-pollination frequency declined with distance (beyond 50 m) from the transgenic source patch (Fig. 1), with significant reduction beginning at a distance 50 m from the transgenic source, falling to 9.3% at 100 m.

Our strategy was to purposefully use two clones that could not intraclonally self-pollinate, our experimental design was set up to gather worst-case scenario data: maximizing potential gene flow. A single clonal type of switchgrass in the pollen source plot and a different single clone as pollen receptor plants theoretically allow a singular mating choice given that switchgrass is putatively and obligate outcrosser. This situation is vastly different than that of a typical commercial field inhabited by a synthetic population of heterogeneous genotypes that would be expected to intermate with each other. Thus, our observed outcrossing rates by distance are likely many-fold higher than that expected in a commercial setting, even if all commercial plants are members of one cultivar. Nonetheless, declining transgenic pollen loads as a function of distance, and the possibility of selfing or apomixis, the former of which has been noted, rarely, in switchgrass [60, 61], may have led to nontransgenic seed production which could have influenced the frequency of observed transgenic progeny. Additionally, if the clone used for our receptor plants has any degree of self-compatibility, it is possible that, along with competitive interplays between the two pollen types and less-than-ideal overlap of floral phenologies, could have also influenced these results, which are similar to those exhibited by the wind-pollinated forage grass *Festuca pratensis* Huds. in a similarly designed, albeit nontransgenic, experiment [62]. In that study, gene flow between the source and receptor plants of *F. pratensis* was 50% at a distance up to 15 m and showed a rapid decrease to 10% at 75 m, and 5% at 155 m [62].

Previously, we have not observed self-compatibility in our experimental clones in the greenhouse or field prior to these experiments. Nonetheless, we have also not isolated it from other genotypes the way they were isolated in these experiments. Thus, it is possible that selfing in these clones were practically below detection before. There have been prior reports of selfing in switchgrass [61, 63] dispelling the view that it is a completely obligate outcrosser. Those findings suggest there can be an occasional breakdown in the self-incompatibility system, which is the most likely explanation of the existence of some nontransgenic progeny. We estimated that at least ca. 90.6% of the pollen cloud was transgenic, with pollen microscopy evidence suggesting it may be even higher. Therefore, the 204 transgenic progeny (22%) from pollinations from pollen sink plants may be an underestimate of gene flow from the pollen-source population. Multiplying 204 by 0.094 (the expected non-transgenic pollen frequency from the transgenic plants) yields an estimated 19 additional progeny likely sired by transgenic plants. Therefore, of the 78% non-transgenic progeny from ‘pollen-sink’ plants, once we subtract the 19 progeny sired by transgenics, we are still left with 739 progeny (77%) coming from apparent self-pollination events. Thus, serendipitously, our results may have inadvertently led to increased evidence of selfing in switchgrass.

Our ability to empirically quantify pollen dispersal distances in switchgrass was arguably only feasible via the use of fluorescent protein tagged pollen source plants. In our case, the majority of pollen produced by our source plants expressed OFP, which distinguished switchgrass pollen from that of other heterospecifics at our field site and made fluorescent screening of pollen traps an efficient endeavor. Other methods utilized for the study of pollen movement have limitations, including destructive analysis or radioactive labels [44, 62, 64–66]. Many researchers have utilized GFP-tagged pollen in transgenic plants to study gene flow [51, 67, 68]. In the present study, OFP was selected as the marker of choice since its expression can be visualized without sample destruction as has been shown with a different OFP variant in tobacco species [69]. The OFP-tagged pollen method is more rapid and convenient than real-time PCR and other such DNA-based screening methods in which DNA extraction is a prerequisite. The OFP tracking technique has great potential as a tool for gene flow and risk assessment studies as well as for post-release monitoring of transgenic plants [70]. The use of OFP and other fluorescent protein markers may also provide a viable option in estimating pollen flow of specific pollen donors in systems with concurrent heterospecific pollen flow and where palynological expertise to identify pollen grains to species is lacking.

Switchgrass pollen is diminutive with a mean diameter range of 36–48 μm (36.02 μm [71], 48.4 μm [46], and 43.7 μm [47]). Switchgrass pollen has short-lived viability after shedding, which also varies under environmental conditions. Greenhouse-grown ‘Alamo’ switchgrass produced pollen that was viable no longer than 20 min under sunny conditions and 150 min under cloudy conditions with a half-life of 4.3 min and 22.9 min; respectively (46). In another study, pollen from greenhouse grown switchgrass plants was exposed to ambient outdoor conditions and viability was completely lost after 60 min for most samples (half-life = 17.3 min) and 100 min for a single sample (47). Short viability periods require pollen to be dispersed quickly for pollination to occur over long distances, yet, depending on wind speeds and other environmental factors, a 60–100 min viability period may allow for pollen dispersal over a large area. Indeed, when switchgrass pollen dispersion was modeled based on 100 min viability, pollen was capable of moving up to 3.5 km in light wind and 6.5 km in stronger wind conditions; the majority of pollen is expected to travel < 200 m (47). In our study we observed a rapid decline in transgenic pollen deposition with increasing distance from the source plot; the majority of pollen deposition occurred within the first 50 m (Fig. 2, Additional file 8). The distance in which the majority of pollen was deposited was nearer to the pollen source than predicted by Ecker et al. [47]; however, the pattern of decline was similar. Our results may differ from the published model because of the secluded nature of our experimental site in which the forested border may have buffeted wind.

Nonetheless, these results are in agreement with pollen dispersal patterns observed in perennial ryegrass (*Lolium perenne*, [72]) where the bulk of pollen deposition occurred within 10 m from the source. Also, meadow fescue (*Festuca pratensis* [62]) pollen frequency declined rapidly within 75 m of the pollen source.

## Conclusions

In a unique small field study in transgenic switchgrass, the first of its kind in which regulated switchgrass was allowed to flower in the field, we collected data on pollen movement and pollen-mediated transgenic hybridization. Over a 3-year period, the majority of pollen movement and cross-pollination occurred in close proximity to the pollen source, yet there was a significant amount of gene flow detected in our outermost sentinel plots (50–100 m) along with some apparent selfing. As U.S. regulatory agencies have set no allowable limits on the amount of adventitious presence for genetically engineered plants, the potentially acceptable limits of transgene flow is unknown in the U.S. for switchgrass and other crop species. If we used the E.U. upper limit of 0.09% [Regulation (EC) No 1830/2003; [73]], it would appear that in our worst-case scenario study, the gene flow observed here would have exceeded E.U. standards. Therefore, we could reasonably expect that at least a 100 m isolation distance would be required between transgenic edges and potential wild or cultivated nontransgenic plants that might receive transgenic pollen. For regulatory and risk assessment purposes, a spatially and genetically heterogeneous design could be useful to predict gene flow from commercial engineered switchgrass. Given the reproduction biology of this perennial species coupled with potentially fitness-enhancing traits (e.g. drought or salt tolerance, rapid growth rate, and disease resistance) [35] imbued via genetic engineering, transgene flow might require mitigation by an engineered bioconfinement system (e.g. selective male-sterility [74] or transgene excision [73].

## Methods

### Transgenic line selection and analysis

Switchgrass (‘Alamo’ ST1 clones) plants were genetically engineered through *Agrobacterium*-mediated transformation, as described in Burris et al. [48], with a vector containing the *pporRFP* (OFP) gene under the control of the maize ubiquitin (*ZmUbi1*) promoter and the hygromycin phosphotransferase (*hph*) selectable marker gene under the control of the rice actin (*OsAct1*) promoter [48]. Transgenic T_1_ hemizygous plants were grown in a greenhouse (16/8 h day/night and 28/22 ° day/night). Transgenic lines were selectively chosen by evaluating T-DNA stable integration and transgene copy number using Southern blot analysis (Additional file 1) methodology as described in Moon et al. [73], with a few changes. Briefly, genomic DNA was extracted of T_0_ transgenic switchgrass events 1–4 and 2–9, along with other *pporRFP* transgenic switchgrass events. Ten micrograms of DNA from each sample was digested with *Bam*HI, which cuts once in the T-DNA, to yield estimates of T-DNA insert number for each line. The blot was probed with ^32^P-labeled *pporRFP* gene fragment. To select the best OFP fluorescent events, visual analysis of OFP fluorescent levels was performed by epifluorescent microscopy for roots and sheaths as described by Burris et al. [48] and pollen (Additional file 2) epifluorescence microscopy [69, 75].

### Field experiment

To examine transgenic pollen movement and pollination between transgenic and nontransgenic switchgrass plants, a 3-year field experiment was performed under USDA APHIS BRS release permits (11-007-106r and 13-046-104r). The field site in Oliver Springs, Tennessee U.S.A. was chosen because of its heavily forested borders that provided a natural barrier from wild or otherwise cultivated switchgrass plants. All experimental plants were transplanted by hand on September 20, 2011, and the total planted area was approximately 0.7 ha. A modified Nelder wheel design [76] consisted of a transgenic pollen-source plot and several pollen-recipient plots placed at 10 m intervals in the four cardinal directions from the pollen-source (Additional file 7). The pollen source and recipient plants had similar growth patterns and flowering times, which assured the highest possibility for transgene flow. The pollen-source plot contained 10 rows planted on 0.3 m centers, and each row contained 10 plants of the same type. Four rows were planted with clonally propagated plants from transgenic line ‘1–4’ (40 plants total) and the remaining six were planted clones from line ‘2–9’ (60 plants total). Post-transplant plant mortality led to the establishment of twenty-six ‘1–4’ plants and fifty ‘2–9’ plants, which remained throughout all experiments. Alamo 2 clones (cultivar ‘Alamo’) were chosen as the pollen-recipients to ensure cross-pollination would not occur between pollen-receptor plots. The long axis of the experiment was set up parallel with prevailing wind direction (eastward). Two receptor plants, 0.6 m apart from each other, were transplanted at distances in 10 m intervals. The northward plots were at 10 m and 20 m, southward and westward at 10 m to 30 m, and eastward from the source plants at10 m to 100 m (Additional file 7). Pollen-receptor plots suffered mortality as well. All plots had at least one plant and plots containing two receptor plants were: west 10 m, south 10 m, east 50 m, east 60 m, east 80 m, and east 90 m.

It is important to note that pollen-donor and pollen-recipient plants are both distinct clonal types originally derived from the cultivar ‘Alamo.’ These clonal types are sexually compatible with each other. However, clones of the same type are putatively not self-compatible. Specifically, pollen-donor plants are putatively not sexually compatible with other pollen-donor plants, and pollen-receptor plants are putatively not sexually compatible with pollen-receptor plants.

### Plant growth and productivity

At the end of each growing season plant height, biomass production, and tiller number were recorded for both pollen-donor and –recipient plants (Table 1). Plant heights were represented by the height of the tallest tiller on each plant. Measurements were taken after panicles were removed for seed collection at the end of the growing season. Although seeds were not collected from pollen-source plants, for consistency panicles were removed prior to height measurement. Biomass measurements were taken as described in Baxter et al. [26] with a few modifications. For each plant, the senesced above-ground biomass was harvested 12.7 cm above the soil, oven-dried at 43 °C for approximately 96 h, and the dry biomass data were taken for each plant. After harvest, tiller numbers were counted for each plant in the field. To ensure plants were grown within typical fertility ranges, soil samples were collected from the source area as well several pollen-receptor plots (east at a distance of 25, 50, 70 and 100 m, and west, north and south at a distance of 15 m). A detailed soil analysis was carried out at the University of Tennessee Soil, Plant and Pest Center in Nashville, Tennessee. Soil properties such as water pH, buffer value, the amount of salts and trace elements were also analyzed (Additional file 3).

### Pollen collection and screening

OFP pollen distribution measurements were taken by placing pollen traps adjacent to each pollen-receptor plot twice in September 2012 and three times in September 2013 for a total of five individual collections. Pollen traps were constructed by covering glass microscope slides (25 mm × 76 mm × 1 mm) with double-sided sticky adhesive tape (3 M136, 3 M Scotch Brand, St. Paul, Minn.) [77]. Pollen traps were mounted horizontally onto vertical wooden stakes with a collection height of 1 m above the soil surface. For each sampling, the slides were exposed for six hour period to collect pollen. OFP pollen frequency was tallied using an epifluorescence microscope (Olympus Reflected Fluorescence system BX51, Olympus Corporation, Shinjuku, Tokyo, Japan) with a TxRed filter set (Texas Red®/Cy3.5, EX-D560/40,BS-595DCLP, EM-D630/60, Olympus Corporation, Shinjuku, Tokyo, Japan). OFP-tagged pollen was imaged using a color digital camera (Olympus QColor 5) with Qcapture imaging software (Q Imaging Corp., Burnaby, Canada). We used Poisson regression analysis (generalized linear mixed model using SAS 9.4; SAS Institute Inc., Cary, N.C.) to examine the relationship of OFP pollen counts and distance from the pollen-source plot. At the time of pollen collection, meteorological data such as temperature, wind speed, humidity, and dew point were recorded using an Ambient weather station located at the Cumberland Forest Unit Headquarters in Oliver Springs, Tenn. (Additional file 6).

### Seed collection, germination and screening

At the end of the 2013 and 2014 growing seasons panicles were collected by hand from pollen-receptor plots and transported back to the laboratory; where seeds were cleaned, segregated by plot and stored at 4 °C for cold stratification. To estimate hybridization frequencies seeds were germinated in an environmental-controlled growth room (16/8 h day/night and 24/22 ° day/night). Seeds were placed in a Petri dishes lined with Whatman® 2 filter paper (Whatman International Ltd., Maidstone, England) and water was added as needed. Once germinated, the resultant seedlings were screened for the presence of OFP through epifluorescent microscopy as described by Burris et al. [48]. One-to-two weeks post-germination seedlings were transferred to potting mix (Fafard® Peat-Lite Mix, Fafard, Inc., Anderson, S.C.) in an environmental controlled growth room (16/8 h day/night and 24/22 ° day/night). To further verify transgenic progeny seedlings were PCR-screened using primers (forward primer: TTTCAAAGCAAAGTGGGGTC; reverse primer: CACCATCTTCAAAGGTCATG) designed to amplify a 302 bp fragment of the *pporRFP* gene. Using a standard protocol genomic DNA was extracted from leaves [78] and PCR was performed using conditions described by Mann et al. [79]. Poisson regression analysis was performed as previously described to evaluate the relationship between distance and transgenic progeny per pollen-recipient plant while taking tiller number per plant into account. For pollen-recipient plots containing two plants, the average number of transgenic progeny was calculated on a per-plant basis. The tallies of F_1_ germinated seedlings, transgenic F_1_ progeny, and tiller tallies were combined for both field seasons.

## Additional files

Additional file 1: Figure S1. Southern blot analysis of T_0_ transgenic switchgrass events containing stable integration of the T-DNA. Blot was probed with ^32^P-labeled *pporRFP* gene fragment. Lanes labeled with ‘2BR’ contain transgenic line genomic DNA samples, lane ‘ST1 control” is the nontransgenic control genomic DNA sample, lane ‘2BR plasmid’ contains the plasmid positive control. Genomic DNA was digested *Bam*HI genomic DNA (10 μg) from each plant sample. (PDF 46 kb)

Additional file 2: Figure S2. Sample of orange fluorescent protein (OFP)-tagged switchgrass pollen from source plants. A), white light, B), TxRed filter, C), merged image. The scale bar is 400 micrometers. (PDF 61 kb)

Additional file 3: Table S1. Soil analysis from field site collected from the pollen-source plot and seven pollen-recipient plots. (PDF 6 kb)

Additional file 4: Figure S3. A sample gel from PCR analysis for the *pporRFP* OFP gene that was performed on F_1_ switchgrass progeny (lanes 1–12) collected from field study pollen-recipient plots. A water only sample (W) and a nontransgenic pollen-recipient parental type (A-; Alamo II) were used a negative controls. A transgenic parental type sample (A+; line 2–9) and a plasmid with the *pporRFP* gene (P+) were used as positive control. A DNA size marker (M) was used to confirm expected band size (302 bp), and blank lanes (B) were used to separate sample types. (PDF 45 kb)

Additional file 5: Table S2. PCR progeny screen of F_1_ seedlings collected from pollen-recipient plots in the field. (PDF 6 kb)

Additional file 6: Table S3. Mean meteorological data collected for the growing seasons of 2012 and 2013 at Oliver Springs, Tennessee. (PDF 6 kb)

Additional file 7: Figure S4. Experimental layout of field study located in Oliver Springs, Tennessee, U.S.A. Transgenic switchgrass (cultivar ‘Alamo’, clone ST1) served as a pollen-source (10 rows × 10 plants) while non-transgenic switchgrass (cultivar ‘Alamo’, clone Alamo 2) plants were placed in pollen-recipient plots planted at 10 m intervals. (PDF 68 kb)

Additional file 8: Average number of orange fluorescent protein (OFP)-tagged switchgrass pollen grains detected as a function of distance in the north, south, and east directions (East, *R* ^2^ = 0.953; North, *R* ^2^ = 0.990; West, *R* ^2^ = 0.930; and South *R* ^2^ = 0.993) Pollen samples were collected in the field during the 2012 and 2013 growing seasons. (PDF 54 kb)
