# Supplementary figures and images for: Pollen-mediated gene flow from transgenic to non-transgenic switchgrass (Panicum virgatum L.) in the field
Source: BMC Biotechnol. 2017 May 2;17:40. doi: 10.1186/s12896-017-0363-4 (PMC5414321; doi:10.1186/s12896-017-0363-4)

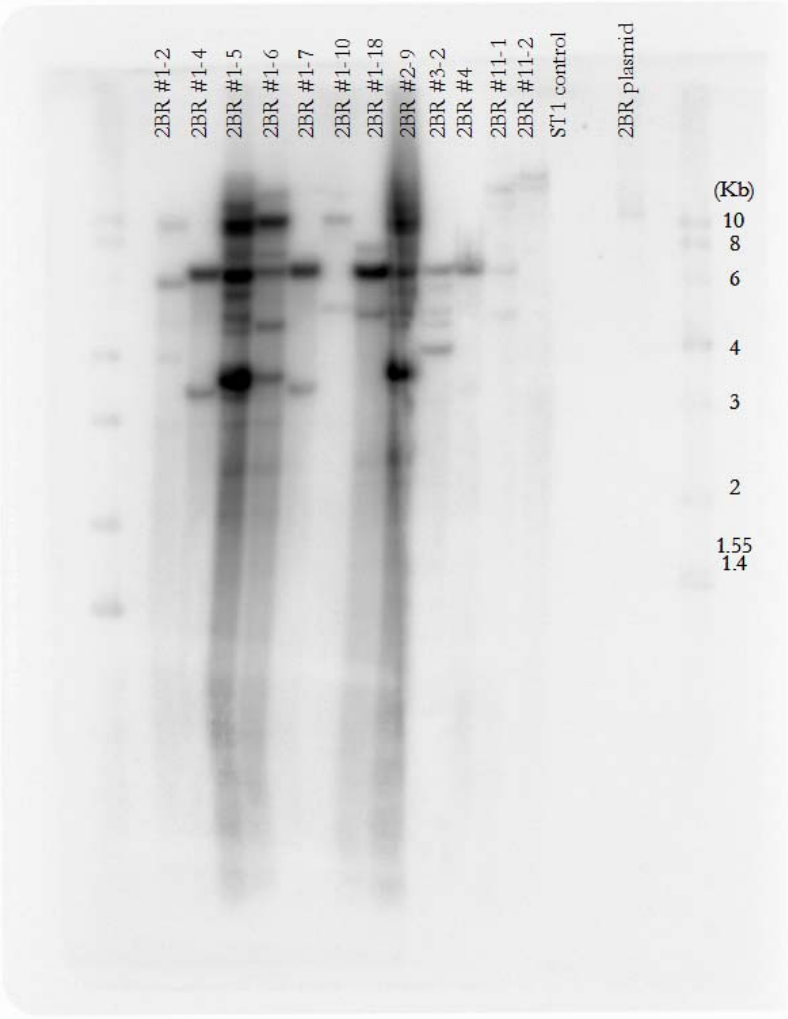

Supplement: Supplementary file 1 — Southern blot analysis of T0 transgenic switchgrass events containing stable integration of the T-DNA. Blot was probed with 32P-labeled pporRFP gene fragment. Lanes labeled with ‘2BR’ contain transgenic line genomic DNA samples, lane ‘ST1 control” is the nontransgenic control genomic DNA sample, lane ‘2BR plasmid’ contains the plasmid positive control. Genomic DNA was digested BamHI genomic DNA (10 μg) from each plant sample. (PDF 46 kb) [file 12896_2017_363_MOESM1_ESM.pdf]

**A**

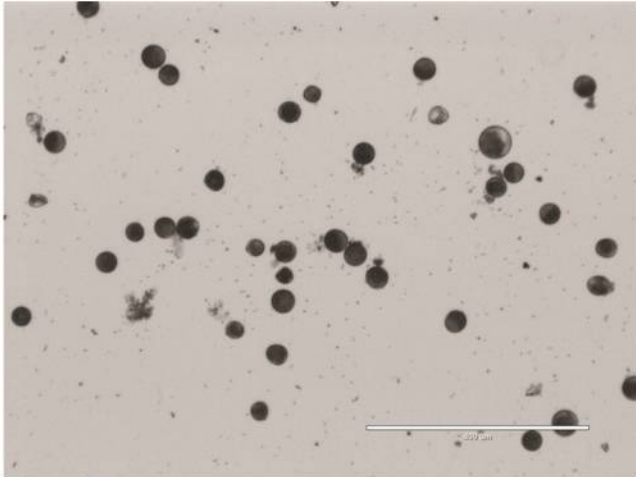

**B**

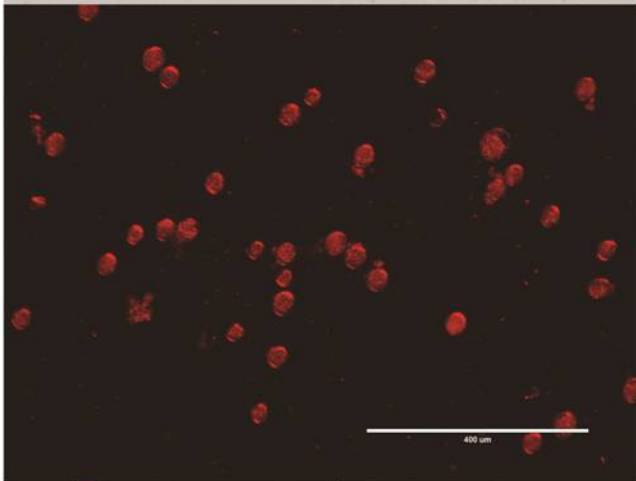

**C**

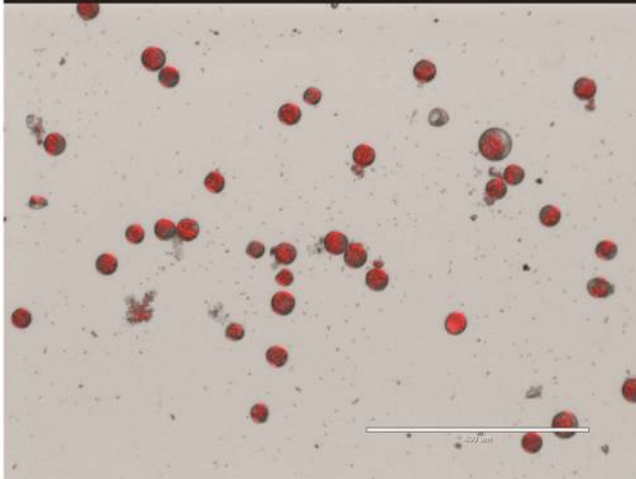

Supplement: Supplementary file 2 — Sample of orange fluorescent protein (OFP)-tagged switchgrass pollen from source plants. A), white light, B), TxRed filter, C), merged image. The scale bar is 400 micrometers. (PDF 61 kb) [file 12896_2017_363_MOESM2_ESM.pdf]

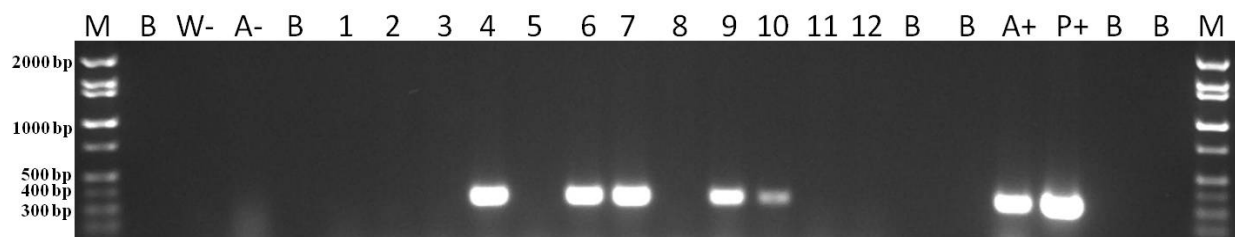

Supplement: Supplementary file 4 — A sample gel from PCR analysis for the pporRFP OFP gene that was performed on F1 switchgrass progeny (lanes 1–12) collected from field study pollen-recipient plots. A water only sample (W) and a nontransgenic pollen-recipient parental type (A-; Alamo II) were used a negative controls. A transgenic parental type sample (A+; line 2–9) and a plasmid with the pporRFP gene (P+) were used as positive control. A DNA size marker (M) was used to confirm expected band size (302 bp), and blank lanes (B) were used to separate sample types. (PDF 45 kb) [file 12896_2017_363_MOESM4_ESM.pdf]

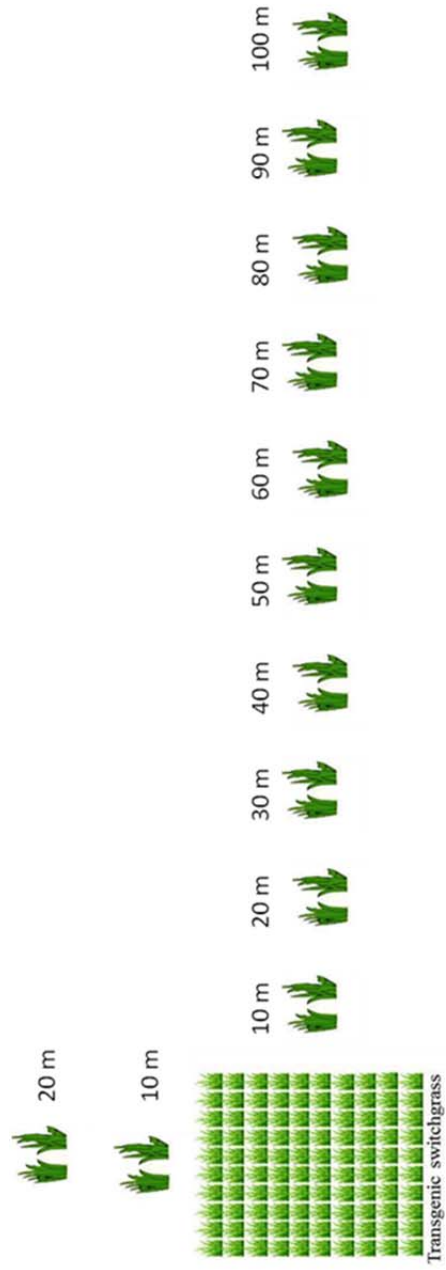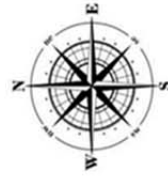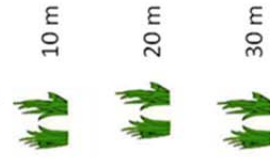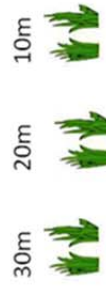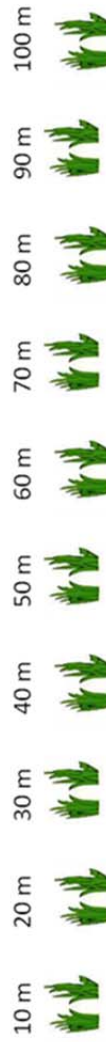

Supplement: Supplementary file 7 — Experimental layout of field study located in Oliver Springs, Tennessee, U.S.A. Transgenic switchgrass (cultivar ‘Alamo’, clone ST1) served as a pollen-source (10 rows × 10 plants) while non-transgenic switchgrass (cultivar ‘Alamo’, clone Alamo 2) plants were placed in pollen-recipient plots planted at 10 m intervals. (PDF 68 kb) [file 12896_2017_363_MOESM7_ESM.pdf]

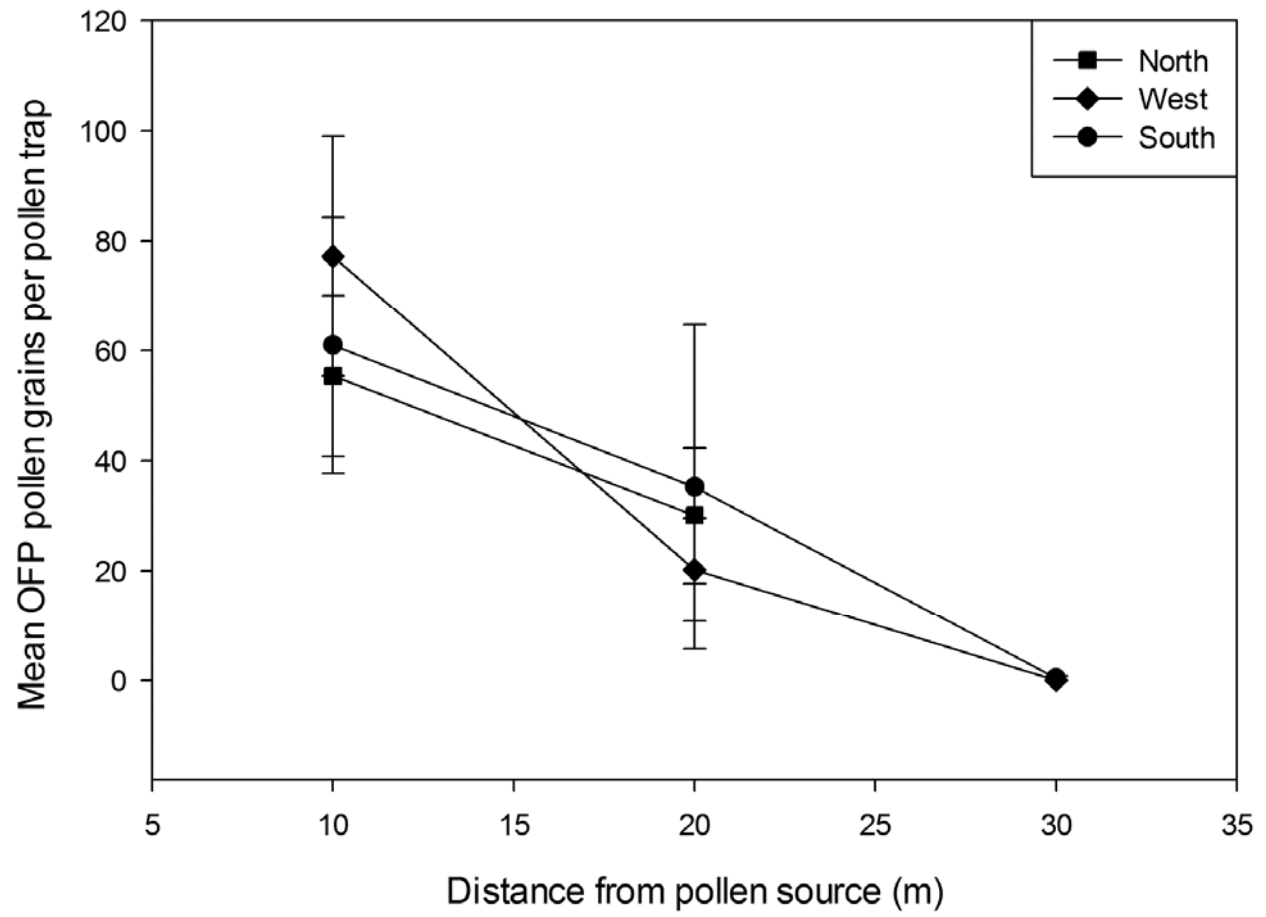

Supplement: Supplementary file 8 — Average number of orange fluorescent protein (OFP)-tagged switchgrass pollen grains detected as a function of distance in the north, south, and east directions (East, R 2 = 0.953; North, R 2 = 0.990; West, R 2 = 0.930; and South R 2 = 0.993) Pollen samples were collected in the field during the 2012 and 2013 growing seasons. (PDF 54 kb) [file 12896_2017_363_MOESM8_ESM.pdf]
