# Supplementary material for: Pollen-mediated gene flow from transgenic to non-transgenic switchgrass (Panicum virgatum L.) in the field
Source: BMC Biotechnol. 2017 May 2;17:40. doi: 10.1186/s12896-017-0363-4 (PMC5414321; doi:10.1186/s12896-017-0363-4)
Supplement: Supplementary file 3 — Soil analysis from field site collected from the pollen-source plot and seven pollen-recipient plots. (PDF 6 kb) [file 12896_2017_363_MOESM3_ESM.pdf]

Table S1. Soil analysis from field site collected from the pollen-source plot and seven pollen-recipient plots.

Soil parameters	Transgenic source plot	North (15 m)	West (15 m)	South (15 m)	East (25 m)	East (50 m)	East (75 m)	East (100 m)
Water pH	5.3	5.2	4.8	4.9	5.1	5.1	5.2	5.1
Buffer value	7.4	7.4	7.1	7.3	7.2	7.2	7.3	7.3
Phosphorus*	5	7	6	5	6	5	10	11
Potassium*	16	53	41	22	29	38	43	70
Calcium*	282	482	144	104	286	410	379	532
Magnesium*	40	84	28	17	61	70	67	70
Zinc*	1.3	4.5	1.3	0.9	2.2	3.9	2.4	4
Copper*	0.6	1.4	0.8	0.4	0.5	0.4	0.5	1.3
Iron*	28	62	38	28	22	22	36	65
Manganese*	7	16	7	8	9 S	8	11	12
Boron*	0.2	0.3	0.2	0.2	0.3	0.3	0.3	0.3
Sodium*	15	16	13	10	14	13	14	15

*Nutrient values are in ppm.
