# Supplementary material for: Pollen-mediated gene flow from transgenic to non-transgenic switchgrass (Panicum virgatum L.) in the field
Source: BMC Biotechnol. 2017 May 2;17:40. doi: 10.1186/s12896-017-0363-4 (PMC5414321; doi:10.1186/s12896-017-0363-4)
Supplement: Supplementary file 5 — PCR progeny screen of F1 seedlings collected from pollen-recipient plots in the field. (PDF 6 kb) [file 12896_2017_363_MOESM5_ESM.pdf]

Table S2. PCR progeny screen of F_1 seedlings collected from pollen-receptor plots during the 2013 and 2014 field seasons.

Field plot	Germinated F_1 seedlings	*pporRFP* PCR positive F_1 seedlings	Transgenic F_1 seedlings (%)
North 10	50	16	32.0
North 20	38	13	34.2
South 10	47	33	70.2
South 20	0	0	0.0
South 30	44	3	6.8
West 10	62	35	56.5
West 20	1	1	100.0
West 30	3	2	66.7
East 10	48	38	79.2
East 20	4	3	75.0
East 30	7	4	57.1
East 40	1	0	0.0
East 50	104	7	6.7
East 60	91	4	4.4
East 70	24	4	16.7
East 80	136	12	8.8
East 90	194	19	9.8
East 100	108	10	9.3
