# Supplementary material for: Pollen-mediated gene flow from transgenic to non-transgenic switchgrass (Panicum virgatum L.) in the field
Source: BMC Biotechnol. 2017 May 2;17:40. doi: 10.1186/s12896-017-0363-4 (PMC5414321; doi:10.1186/s12896-017-0363-4)
Supplement: Supplementary file 6 — Mean meteorological data collected for the growing seasons of 2012 and 2013 at Oliver Springs, Tennessee. (PDF 6 kb) [file 12896_2017_363_MOESM6_ESM.pdf]

Table S3. Mean meteorological data collected for the growing seasons of 2012 and 2013 at Oliver Springs, Tennessee, USA.

**Weather**	**Mean**
Dew point (Celsius)	15.7
Wind speed (km/h)	5.2
Daily low temperature (Celsius)	18.3
Daily high temperature (Celsius)	25
Precipitation (cm)	7.9
Humidity - morning (percentage)	90
Humidity - afternoon (percentage)	57
Humidity recorded (percentage)	52.7
Sunshine (percentage)	62
Cloudy days - clear of clouds (percentage)	35
Cloud days - partly cloudy (percentage)	25
Cloudy days - cloudy days (percentage)	40
Cloudy days - days with precipitation (percentage)	30
